# Predictive coding is a consequence of energy efficiency in recurrent neural networks

**DOI:** 10.1016/j.patter.2022.100639

**Published:** 2022-11-23

**Authors:** Abdullahi Ali, Nasir Ahmad, Elgar de Groot, Marcel Antonius Johannes van Gerven, Tim Christian Kietzmann

**Affiliations:** 1 Donders Institute for Brain, Cognition and Behaviour, Radboud University, Nijmegen, the Netherlands; 2 Institute of Cognitive Science, University of Osnabrück, Osnabrück, Germany; 3 Department of Experimental Psychology, Utrecht University, Utrecht, the Netherlands

**Keywords:** predictive coding, recurrent neural networks, energy efficiency, brain-inspired machine learning

## Abstract

Predictive coding is a promising framework for understanding brain function. It postulates that the brain continuously inhibits predictable sensory input, ensuring preferential processing of surprising elements. A central aspect of this view is its hierarchical connectivity, involving recurrent message passing between excitatory bottom-up signals and inhibitory top-down feedback. Here we use computational modeling to demonstrate that such architectural hardwiring is not necessary. Rather, predictive coding is shown to emerge as a consequence of energy efficiency. When training recurrent neural networks to minimize their energy consumption while operating in predictive environments, the networks self-organize into prediction and error units with appropriate inhibitory and excitatory interconnections and learn to inhibit predictable sensory input. Moving beyond the view of purely top-down-driven predictions, we demonstrate, via virtual lesioning experiments, that networks perform predictions on two timescales: fast lateral predictions among sensory units and slower prediction cycles that integrate evidence over time.

## Highlights

• Neural networks are optimized for energy efficiency in predictable environments
• Trained networks exhibit hallmarks of predictive coding as an emergent phenomenon
• Energy-efficient networks separate into subpopulations of prediction and error units
• Lesion studies indicate two types of prediction: fast and slow

## The bigger picture

In brain science and beyond, predictive coding has emerged as a ubiquitous framework for understanding sensory processing. It postulates that the brain continuously inhibits predictable sensory input, sparing computational resources for input that promises high information gain. Using artificial neural network models, we here ask whether hallmarks of predictive coding can arise from other, perhaps simpler principles. We report that predictive coding naturally emerges as a simple consequence of energy efficiency; networks trained to be efficient not only predict and inhibit upcoming sensory input but spontaneously separate into distinct populations of “error” and “prediction” units. Our results raise the intriguing question which other core computational principles of brain function may be understood as a result of physical constraints posed by the biological substrate and highlight the usefulness of bio-inspired, machine learning-powered neuroscience research.

## Graphical abstract

**Figure:**
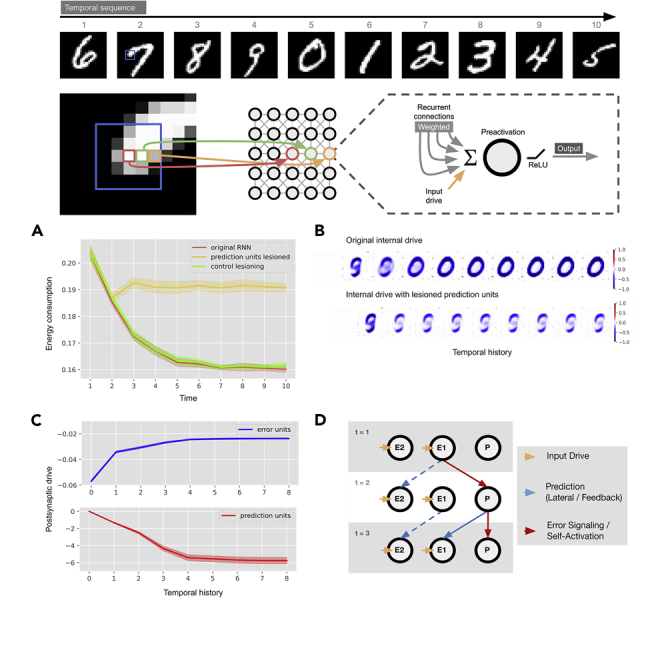

## Teaser

Connecting the computational principles of brain function to the physical constraints of the brain is important for understanding neural information processing. This is demonstrated by showing that the hallmarks of the predictive coding framework can emerge in a recurrent neural network model that is constrained in its energy usage.

## Introduction

In computational neuroscience and beyond, predictive coding has emerged as a prominent theory for how the brain encodes and processes sensory information.^1^^,^^2^^,^^3^^,^^4^ It postulates that higher-level brain areas continuously keep track of the causes of lower-level sensory input and actively inhibit incoming sensory signals that match expectation. Over time, the brain’s higher-level representations are shaped to yield increasingly accurate predictions that, in turn, minimize the surprise the system encounters in its inputs. That is, the brain creates an increasingly accurate model of the external world and focuses on processing unexpected sensory events that yield the highest information gain.

Adding to the increasing, albeit often indirect, experimental evidence for predictive coding in the brain,^5^^,^^6^^,^^7^^,^^8^^,^^9^^,^^10^^,^^11^^,^^12^^,^^13^^,^^14^^,^^15^^,^^16^^,^^17^ computational modeling has investigated explicit implementations of predictive coding, indicating that they can reproduce experimentally observed neural phenomena.^3^^,^^18^^,^^19^^,^^20^ For example, Rao and Ballard^18^ demonstrated that a hierarchical network with top-down inhibitory feedback can explain extra-classical field effects in primary visual cortex (V1). In addition, deep neural network architectures wired to implement predictive coding have been shown to work at scale in real-world tasks,^21^^,^^22^^,^^23^^,^^24^^,^^25^ adding more support for the computational benefits of recurrent message passing.^26^^,^^27^^,^^28^^,^^29^^,^^30^ The common rationale of predictive coding-focused modeling work is to test its computational and representational effects by hardwiring the model circuitry to mirror hierarchical and inhibitory connectivity between units that drive predictions and units that signal deviations from said predictions (also called sensory or error units).

Contrasting this approach, here we ask a different question: can computational mechanisms of predictive coding naturally emerge from other, perhaps simpler computational principles? In search for such principles, we take inspiration from the organism’s limited energy budget and previous established links between prediction and energy efficiency.^31^^,^^32^^,^^33^^,^^34^^,^^35^^,^^36^ Expanding previous work on efficient coding and sparse coding,^37^^,^^38^^,^^39^^,^^40^^,^^41^^,^^42^^,^^43^ we subject recurrent neural networks (rate-based recurrent neural networks [RNNs]) to predictable sequences of visual input and optimize their synaptic weights to minimize the largest sources of energy consumption in the mammalian cortex: action potential generation and synaptic transmission.^44^^,^^45^^,^^46^^,^^47^^,^^48^^,^^49^ We then test the resulting networks for phenomena typically associated with predictive coding. We compare the energy budget of the networks with two baseline models trained with more conventional efficient coding objectives, search for inhibitory connectivity profiles that mirror sensory predictions, and explore whether the networks automatically separate their neural resources into prediction and error units.

## Results

**Figure 1:**
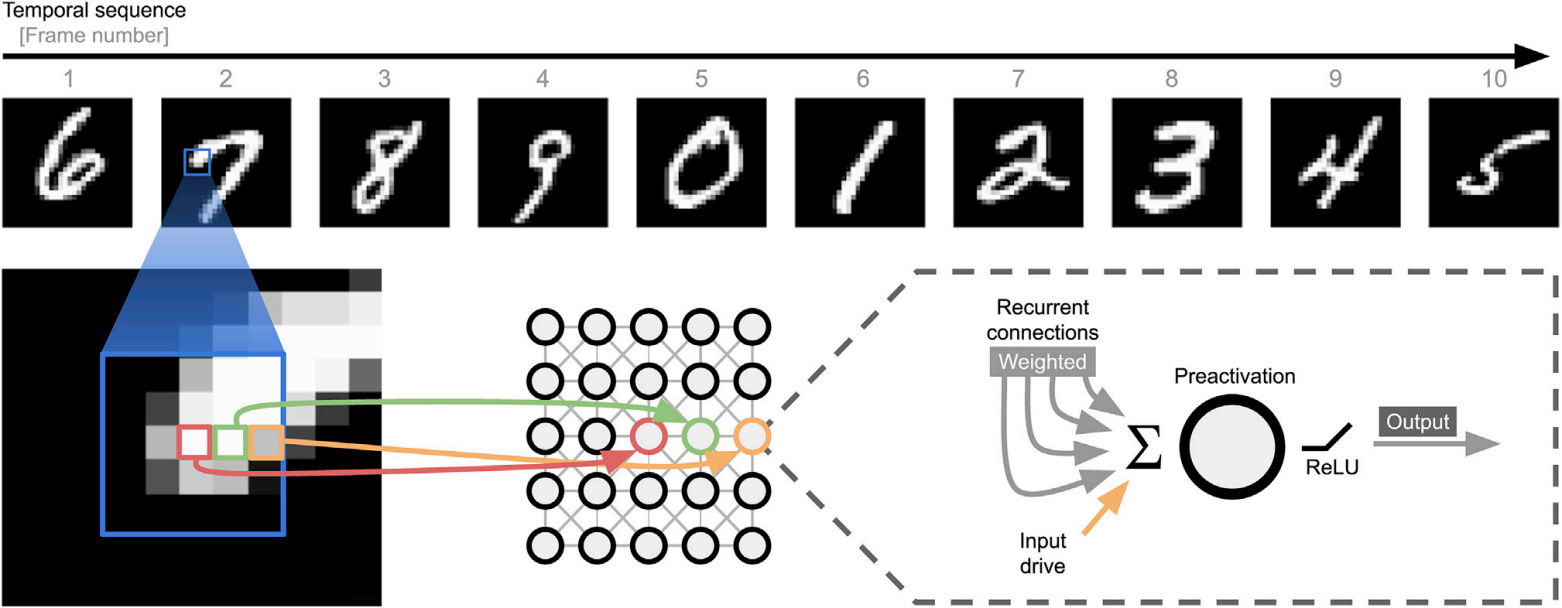
Overview of the model architecture and input pipeline The top part of the figure shows an example input sequence fed to a recurrent network. In each run, 10 randomly sampled MNIST digits are presented in ascending order to the network (with wraparound after digit 9). Each input pixel drives exactly one unit of the RNN. The preactivation of each RNN unit is computed from the recurrent input, provided by other networks units, as well as the input drive determined by the intensity of the pixel to which it is connected. The output of the network units is determined by taking the rectified linear unit (ReLU) activation function of the preactivations. Only the recurrent connections in the network are learned.

To better understand whether, and under which conditions, predictive coding may emerge in energy-constrained neural networks, we first trained a set of RNNs on predictable streams of visual input. For this, we created sequences of handwritten digits taken from the MNIST dataset,^50^ iterating through 10 digits in numerical (category) order. Starting from a random digit, sequences wrapped around from nine to zero. The image sequences shown were deterministic in the sequence of digit categories and therefore (increasingly) predictable when the position in the sequence was extracted by the network. Such predictability of sensory input from the environment is a central assumption of the predictive coding framework because prediction is the primary mechanism through which the framework reduces uncertainty. A temporal autocorrelation of sensory signals is evident in the real world and the consequential assumption of related coding principles, such as slowness^51^ and temporal stability.^52^ Although this work considers the simpler case of predictable sequences of categories, we expect that temporal autocorrelation in natural video data, which is similarly predictable, will yield very similar results. The visual input to each network unit was stimulus dependent, with each unit receiving an input that mirrored its corresponding pixel intensity (i.e., 784 network units corresponding to 784 input pixels from MNIST). These visual inputs were not weighted with modifiable parameters, preventing the models from learning to ignore the input as a shortcut to a solution for energy efficiency. Crucially, because we are using an RNN, the next image in a sequence can be predicted. In other words, we trained the neural network not just to encode a set of images but the particular sequence that was conserved over training. We hypothesized that the models would learn to use their recurrent connectivity to counteract the input drive where possible and, therefore, to minimize their energy consumption. Energy costs were defined to arise from synaptic transmission and action potentials, mirroring the two most dominant sources of energy consumption in the mammalian cortex.^44^^,^^45^^,^^46^^,^^47^^,^^48^^,^^49^ Figure 1 shows an overview of the experimental pipeline. More details are provided under “Data generation” and “RNN architecture and training procedure.”

### Preactivation as a proxy for the energy demands of the brain

**Figure 2:**
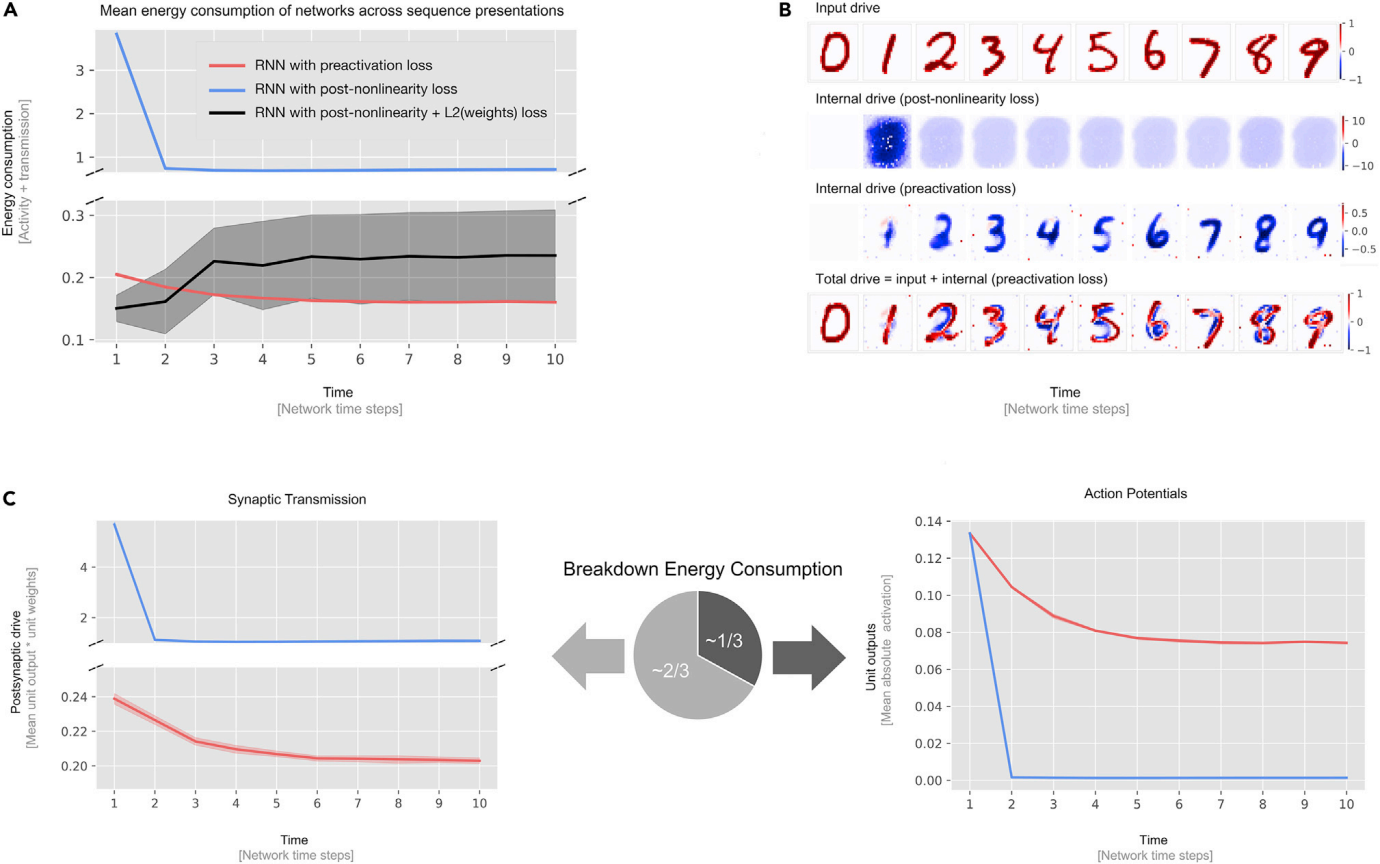
Networks trained with preactivation minimize their energy consumption and learn to predict their input (A) Evolution of the “energy consumption” of trained networks. The models trained to minimize preactivation are compared with control models that are trained to minimize network output (post non-linearity) with an L1 norm on the network output plus an L2 norm on the network weights. Each plot is shown with 95% CIs, bootstrapped across 10 network instances. Networks trained to minimize preactivation achieve lower energy when presented with sequences. (B) Visualization of RNN unit drives and activity for an example sequence. Units are organized according to their input pixel location. Excitatory signals are depicted in red, and inhibitory signals are depicted in blue. The darker the color, the stronger the signal. The sequence images shown were not used for training. The observable improvements in inhibitory trained network drive indicate that the RNN trained for preactivation minimization integrates sensory evidence over time, leading to better internal knowledge of the state of the world and more targeted inhibitory predictions. This is in stark contrast with the drive from the network trained with post-non-linearity loss, which displays imprecise inhibition that also does not improve over the sequence. If we look at the total drive (input + internal drive; i.e., preactivation), then we see that the internal drive is slightly off in matching the input. This is likely a result of the network not being able to match variation of individual writing styles in a particular digit category. (C) Breakdown of energy consumption into activity and synaptic transmission for networks trained with preactivation versus networks trained on activation only. The networks trained with activation outperform the preactivation networks by a substantial margin but do so by incurring a massive cost in synaptic transmission, leading to an overall increase in synaptic transmission.

Sparse coding is often implemented with an L1 sparsity constraint alongside a reconstruction objective on the unit outputs; i.e., networks are optimized to reduce the overall unit activity post non-linearity by having as few units active as possible while reconstructing the input as closely as possible.^39^ Here we take a different approach by making energy minimization the target objective instead. However, given that synaptic transmission contributes considerable energy cost alongside action potentials, a modified learning objective is required to approximate total energy consumption; for example, minimization of unit outputs alone could be implemented via unreasonably strong inhibitory connections, which themselves might increase the overall energy budget. To solve this issue, we propose to instead train energy-efficient RNNs by minimizing absolute (L1) unit preactivation. In real neuronal networks, one can regard the preactivation as presynaptic input; in other words, the summed afferent input from all other neurons. This has two desired properties: first, it drives unit output toward zero, mirroring minimization of unit firing rates (dependent naturally on the form of activation function used). Second, we show that this objective also leads to minimal synaptic weight magnitudes in cases of noise in the system, mirroring an overall reduction in synaptic transmission (see the supplemental text and Figure S5 in the supplemental information for a theoretical derivation and visualization of why this is the case). We consider this link between a biologically realistic notion of energy efficiency and synaptic weight magnitude an important first result of this paper. Optimizing for minimal preactivation, we trained 10 network instances with different random weight initializations.^53^ To empirically assess whether minimizing preactivation leads to better energy consumption outcomes, we need to establish that minimizing preactivation indeed minimizes activity and synaptic transmission and show that it does this better than other, more standard efficient coding objectives. With this in mind, we devised two candidate baseline models: a more conventional model, where the unit outputs (post non-linearity) are minimized, and a model where the unit outputs and weights are minimized. Inclusion of these candidates allows us to explore how overemphasis on minimizing unit outputs can affect synaptic transmission and how to penalize the weights to correct this overemphasis. The results of this comparison can be found in Figure 2A. As postulated, the preactivation models outperform the models that minimize unit output alone.

If we break down the network’s energy consumption into contributions from activities and synaptic transmissions, we see that this divergence is a result of the model minimizing unit output at the cost of high synaptic transmission. Although the preactivation model has higher network activity, it performs much better in terms of synaptic transmission and, hence, in terms of overall energy efficiency. This is due to the fact that the preactivation model penalizes excessively large (negative) weights because they would massively increase synaptic transmission even when that achieves low unit outputs. What would happen if we added a penalty on the weight magnitudes? We tested networks with L2 penalties on the weights and found that these networks can be competitive in terms of energy demands. However, they achieve this through broad inhibition in a similar fashion to the networks trained on the outputs alone (see Figure S1 for a more in-depth breakdown).

The results in Figure 2A are based on a point estimate of energy demands in an animal brain model; i.e., biological assumptions about the relative energy cost of action potentials and synaptic transmission. Our estimate is in line with biological estimates for the mammalian cortex, which commonly estimate the total synaptic transmission to induce higher energy costs than action potentials.^44^^,^^45^^,^^46^^,^^47^^,^^48^^,^^49^ The results do not depend on the particular weighting of action potentials and synaptic transmission and can be changed to increase the impact of action potentials (from zero to 10-fold) on the system without significantly changing the results. In short, minimizing unit preactivation consistently outperformed the minimization of unit output (L1 on the unit activity) in terms of total energy budget.

### RNNs trained for energy efficiency learn to predict and inhibit upcoming sensory signals

Having established that the trained RNNs successfully reduce their energy consumption over time, we confirmed targeted inhibition of predicted sensory input, a hallmark of predictive coding. This was accomplished by separately visualizing the input drive (i.e., the unit activation driven by sensory input) and the network drive (i.e., the unit activation because of network-internal connectivity alone). Figure 2B depicts the aforementioned drives elicited from an example sequence sampled from test data (see “RNN architecture and training procedure” for more details). Two observations with respect to the network trained with preactivation loss are of importance in this example. First, the network trained with preactivation loss clearly predicts and inhibits the upcoming digit category. Second, as time progresses, the network is able to integrate information, leading to better knowledge of the current sequence position and more targeted inhibition (e.g., the inhibition of later digits appears more strong/targeted than earlier predictions that appear weaker/smoother). Better predictions, in turn, result in progressively lower network activity, as quantified previously. This observation follows from the shape of our loss function under “RNN architecture and training procedure.” Replacing $p_{t}$ with $-p_{t}$ in Equation 1 will yield a prediction error loss. This tight link has been established before.^31^^,^^32^^,^^33^^,^^34^^,^^35^^,^^36^ In our work, the link can be explained in the following way: the preactivation allows “predictive inhibition” while forcing the network weights to be as small possible so that irreducible noise has as little impact as possible on the network activity. We refer the reader to thesupplemental information for a derivation of why this is the case. Another important observation is that the network trained on unit outputs alone is not able to generate sharp predictions, showcasing that this objective is not a good proxy for energy consumption. In addition to the expected emergence of prediction in our model, this pattern of results is in line with the hierarchical predictive coding framework, in which feedback from higher-order areas is subtracted from sensory evidence of lower areas. However, a hierarchical organization was not imposed in our RNNs. Rather, it emerges because of optimization of the networks for energy efficiency.

### Separate error units and prediction units as an emergent property of RNN self-organization

Our previous results demonstrate that RNNs, when optimized to reduce their energy consumption (action potentials and synaptic transmission), exhibit key phenomena associated with predictive coding. Predictive coding emerges, without architectural hardwiring, as a result of energy efficiency in predictable sensory environments. A second key component postulated by the predictive coding framework is the existence of distinct neural populations that provide sensory predictions or carry the deviations from such predictions; i.e., prediction and error units.^54^ Given the non-hierarchical, non-constrained nature of the current setup, we next wanted to determine whether a similar separation could also be observed in our energy-efficient networks.

If the networks had indeed developed prediction units, we could potentially identify them by looking at biases in their (median) preactivation at the latter time points of the sequence (i.e., the time when network dynamics should be most stable). The rationale is as follows: because the networks are trained to minimize absolute preactivation, we would expect that the median preactivation for a unit is close to zero. However, if a units’ median preactivation is significantly different from zero, then that would imply that this unit has some functional role in the network, which, in this case, is prediction. Following this rationale, we identified candidates for prediction units by calculating the median preactivation of each unit across multiple instances of each digit category during presentation of the final element of the sequences.

Units were labeled to be potentially predictive when, for any one class, the 99% confidence interval (CI) around the median preactivation did not include zero. That is, they showed a consistently deviation from a “zero activity” profile, which would have been inhibited if it did not serve a functional role in the overall objective of energy efficiency. See “Determination of prediction and error units” for details about the exact procedure for categorizing units and calculation of CIs for the median preactivation and median absolute deviation (MAD) around the median preactivation.

**Figure 3:**
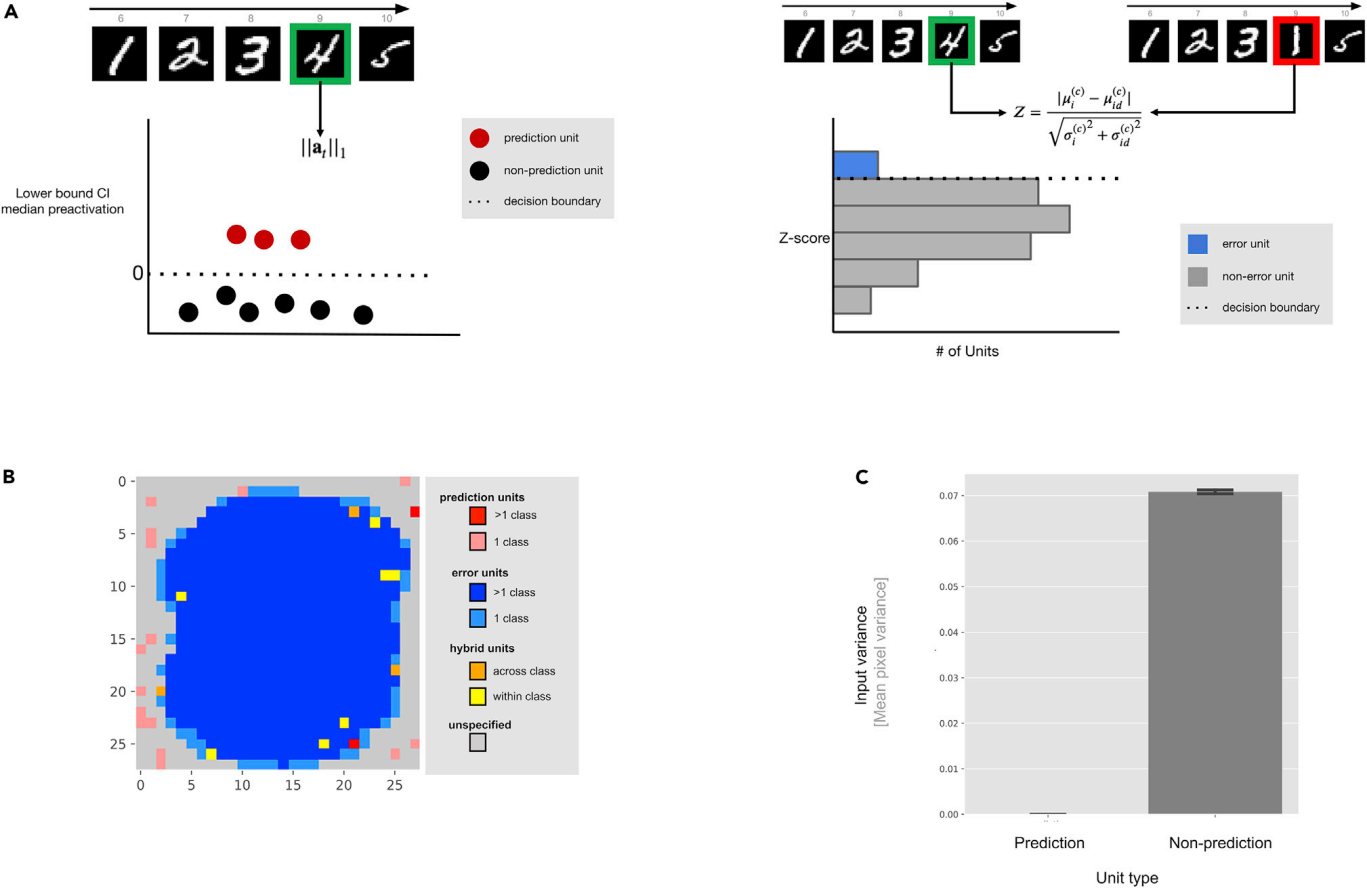
Trained RNNs exhibit populations of prediction and error units (A) Illustration of how prediction and error units are determined. The left illustration shows the process of prediction error selection. For each unit, the median preactivation and a CI around that median are determined (for a particular class). If zero is not within the CI, then our method identifies the unit as being predictive (for that class). The right illustration shows how error-signaling units are determined. The network is subjected to two sets of sequences. In one set, the sequence contains a distractor digit that breaks the order in the sequence. If a unit’s (class-wise) mean response differs significantly between the two sets of sequences (99% CI), then we label them as error signaling. (B) Topographical distribution of unit types in MNIST space. Gray units have no functional role in the network. Units that only have one functional role (i.e., prediction or error signaling) are stained red or blue, respectively, with the particular shade depending on whether the units respond to a single class or multiple classes. Units that are identified as performing prediction and error signaling (i.e., hybrid) are stained orange or yellow, depending on whether they fulfill both roles in a class or across classes. (C) Input pixel variance as a function of unit type. Prediction units emerge in areas with low pixel variance (error bars shown with 95% CI).

To identify error-signaling units, a different approach is necessary. If the networks developed error units, then they could be found by analyzing the unit responses during presentation of surprising inputs. In this light, we constructed an additional set of sequences in which the digit at the second-to-last time point is swapped out with a random digit from another digit class. Response distributions of each network were constructed for these surprising sequence events and compared with the response distribution for normal sequences. Units were labeled to be potentially error-signaling, if the means of the respective distributions were statistically different (99% CI). An illustration of this analysis is shown in Figure 3A. When all prediction units are collected (16.7±0.48 units, depending on the particular network instance), they occupy a different topographic part of the RNN relative to the error units (see Figure 3B, which shows the general distribution of postulated prediction and error units across all 10 digit categories). Units with consistent non-zero activation (i.e., the postulated prediction units) reside in locations that are in low-pixel variance areas typically not strongly driven by sensory input, as evident in the structure of the MNIST dataset. Experiments on CIFAR-10(Figure 5B) with networks with and without additional latent resources confirm that prediction units tend to emerge in areas of lower input variance. The peripheral locations of the putative prediction units occupy parts of the input space that are typically black (i.e., unused) or lower variance when such “empty” input space does not exist. Such secondary use of neural resources that would otherwise remain unused is in line with neuroscientific evidence for cortical plasticity and reorganization.^55^^,^^56^ In addition to prediction and error units, we also observe a population of units that behave as prediction and error units; i.e., hybrid units. Investigating the role of these (few) units in the network dynamics will be the focus of future work. We refer the reader to Figure S4 for a visualization of the topography of an untrained network.

Until now, we have not established a causal effect or functional role of the postulated prediction units in the network dynamics. This will be addressed in depth in the next section.

### Prediction units integrate evidence over time for more targeted inhibition

**Figure 4:**
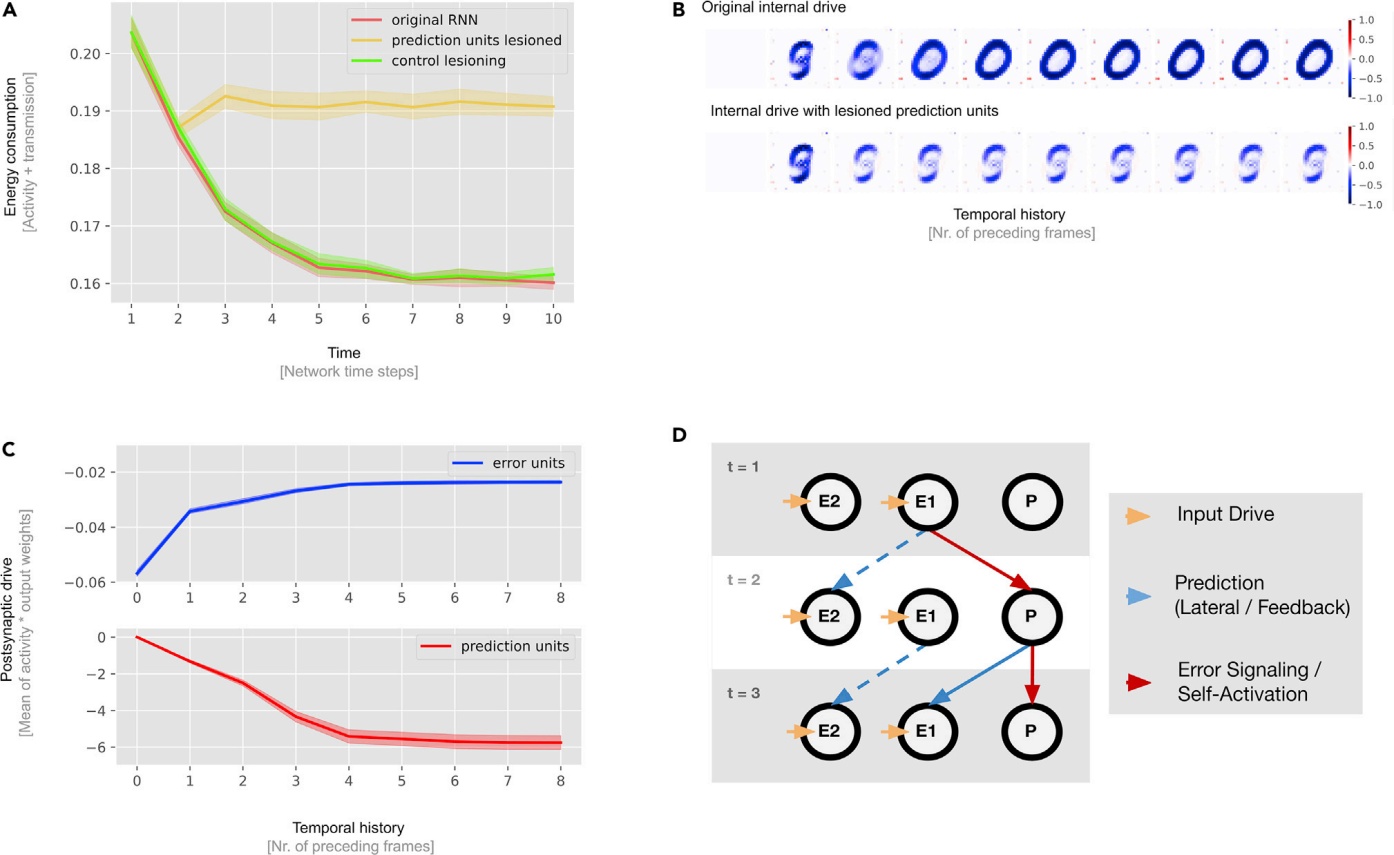
Lesion studies reveal the causal role of prediction units (A) Mean energy consumption of normal RNNs (red line), prediction unit lesioned RNNs (yellow line), and control lesioned RNNs (green line). In contrast to regular RNNs, the prediction unit lesioned RNNs do not reduce their energy consumption throughout the sequence, indicating that they are not capable of integrating evidence over time. Control experiments demonstrate that lesioning non-prediction units instead of prediction units has little effect on network dynamics (green line). From this, we can conclude that the prediction units have a unique functional role in prediction. Mean energy consumption is shown with 95% CIs, bootstrapped across 10 network instances with replacement. (B) Internal drive $\left(p_{t}\right)$ in the RNN for the “0” digit category as a result of varying temporal history prior to stimulus presentation. Each square shows the internal drive of the network following various sequence lengths prior to the stimulus of interest; e.g., the sixth image displays the internal drive after the sequence “5-6-7-8-9” was presented to the network. The first row shows the predictions of a normal RNN, and the second row shows the result of lesioning RNN prediction units. The internal drive of the lesioned network does not develop better predictions with longer sequence lengths. (C) Prediction and error units have different postsynaptic drive dynamics. Initially, error units inhibit the network, but this inhibitory drive diminishes as prediction units take over. This is in line with the hypothesis of two mechanisms of prediction that operate on different timescales. (D) Illustration of how the two predictive mechanisms might interact in a toy network of two error units (E1 and E2) and one prediction unit (P). Initial input drive excites an error unit, which then inhibits neighboring error units (lateral inhibition) and excites adjacent prediction units. In the next time step, the prediction unit inhibits its target error-signaling units (feedback inhibition). In this example, E1 one might be a target because its activity persists in the next time step.

To explicitly test their functional role in RNN dynamics, we performed virtual lesion experiments with the candidate prediction units, as identified in the previous section. As shown in Figure 4B, top row, an unlesioned RNN is capable of more fine-grained predictions with increasing sequence length exposure. In stark contrast to this, networks with lesioned prediction units (Figure 4B, bottom row) remain fixed at the initial and immediate prediction and lose the ability to integrate evidence over time. This prediction resembles the median of all images of the MNIST data (Figure S3), which the is the optimal solution when the network cannot integrate category-specific information. Figure S2 shows more lesion experiments targeting other digit categories. Next we quantified the effects of lesioning prediction units (Figure 4A). After an initial reduction in energy consumption (investigated in depth in the next section), the lesioned networks fail to reduce their activity over time (yellow line). As a control experiment, we lesioned the same number of units from outside the population of identified prediction units rather than prediction units (green line). This had a minimal effect on network energy consumption, verifying the special role of prediction units in overall network dynamics. See “procedure for prediction unit lesions and control lesions” for a more detailed description of the lesion procedures.

### Two distinct inhibitory mechanisms work at different timescales

As can be surmised from Figures 4A and 4B, the energy consumption of the network drops from the first to the second timestep by means of an imprecise but category-specific inhibition. This network behavior is of interest because potential prediction loops, from input to prediction units and back, require two time steps to come into effect (one to activate prediction units and one for these prediction units to provide some feedback). To confirm this observation, we looked at how prediction and error units drive other units with increasing temporal history (Figure 4C). We observe that their postsynaptic drive dynamics show opposite trends. The initial inhibitory drive results from the error units, but as temporal history increases (i.e., more of the sequence is observed), their inhibitory drive weakens. Contrary to this, prediction units show a continual increase in inhibition as more of the sequence is observed, followed later by a saturation period. This suggests that the observed predictions in time step two are rather driven by more immediate lateral connections among error units; to our knowledge, a mode of predictive coding not considered previously. We observe two different modes of predictive inhibitions: one operating at a faster timescale among error units and one on a slower timescale involving prediction units with the potential to integrate evidence over time. See Figure 4D for a schematic of how these two mechanisms of predictions may operate in the network.

### Replication of main results: CIFAR-10

**Figure 5:**
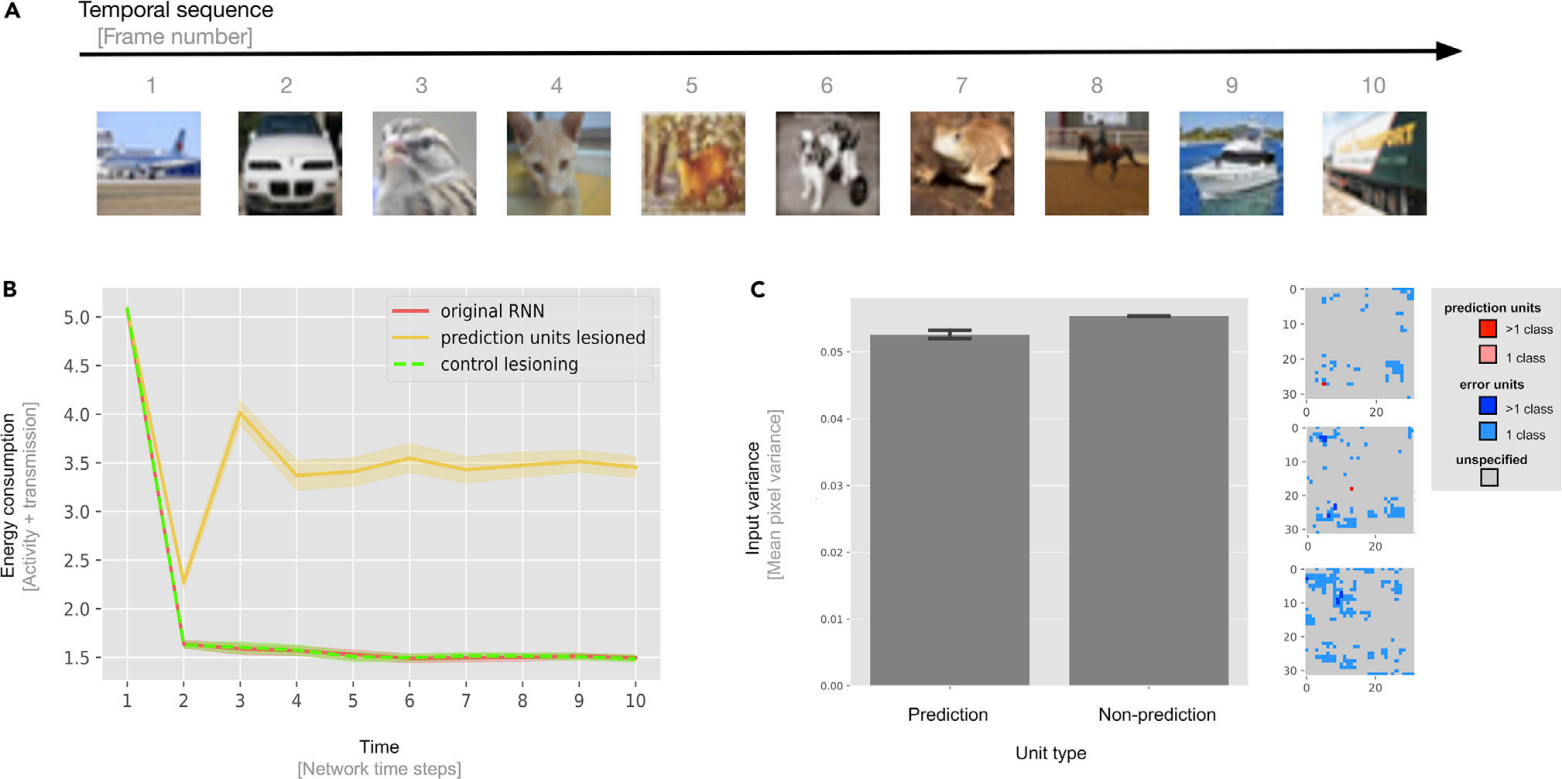
Results generalize to CIFAR-10 (A) Example input sequence fed to the network. In each run, 10 randomly sampled CIFAR-10 images are presented in ascending order to the network (with wraparound after image class 9). (B) Mean energy consumption of normal RNNs (red line), prediction unit lesioned RNNs (yellow line), and control lesioned RNNs (green line). In contrast to regular RNNs, the prediction unit lesioned RNNs do not reduce their energy consumption throughout the sequence, indicating that they are not capable of integrating evidence over time. Control experiments demonstrate that lesioning non-prediction units instead of prediction units has little effect on network dynamics (green line). From this, we can conclude that the prediction units have a unique functional role in prediction. Mean energy consumption is shown with 95% CIs, bootstrapped across 10 network instances with replacement. (C) Prediction units have mildly lower input variance. Left: input pixel variance as a function of unit type. Prediction units tend to emerge in areas with low pixel variance. Error bars are shown with 95% CIs. Right: topographical distribution of populations in CIFAR-10 space across the three color channels (R, G, and B). Gray units have no functional role in the network. Units that only have one functional role (i.e., prediction or error signaling) are stained red or blue, respectively, with the particular shade depending on whether the units respond to a single class or multiple classes. No hybrid units were identified in CIFAR-10.

Having established that predictive coding can emerge as a result of training RNNs for energy efficiency, we tested the generality of our results by testing on a separate dataset with more realistic image statistics (e.g., full image coverage, unlike MNIST, in which information is predominantly present in central pixels). In particular, we performed experiments as before but used sequences of CIFAR-10 images (Figure 5A). Replicating our previous results, we again observe the emergence of two distinct populations of units, with lesions to prediction units exhibiting strong effects on predictive performance and energy consumption of the network (Figure 5B). One notable difference is that that we observe far fewer prediction and error units in CIFAR-10 (1.8±0.42 and 179.2±137.77 prediction units on average) than in MNIST (16.7±0.48 and 552±1.7 prediction and error units, respectively, on average) despite the networks being bigger (784 units versus 3072 units). We attribute this to differences in the pixel variance profile of the data for CIFAR-10 versus MNIST, as can be seen in Figures 3B, 3C, and 5C. Prediction units tend to congregate in areas with lower pixel variance. MNIST clearly separates into high and low variance pixels, whereas input variance in the case of CIFAR-10 is much more varied because of not having perpetually dark pixels. In MNIST, the prediction units appear in areas with low variance (the edges), whereas error units appear in areas with high pixel variance (the center). In CIFAR-10, although less pronounced because of the less extreme fluctuations in pixel variance, we observe a similar trend. We also conducted analyses of models with additional “latent” (i.e., excess) units that are not driven by any input. In these models, the prediction units consistently emerged in the latent part of the network, which, by architectural design, is part of the network with the lowest input variance. This pattern could be explained by the fact that error-signaling units seem to be characterized by shifts in response distributions, whereas prediction units benefit from low variance to slow down their dynamics and respond to fluctuations caused by error signaling.

The networks are still able to minimize preactivation and reproduce the lesioning results seen in the MNIST dataset (Figure 5B). See “data generation” for specific details about the CIFAR-10 dataset.

## Discussion

Here, we demonstrate that RNN models, trained to minimize biologically realistic energy consumption (action potentials and synaptic transmissions), spontaneously develop hallmarks of predictive coding without the need to hardwire predictive coding principles into the network architecture. This opens up the possibility that predictive coding can be understood as a natural consequence of recurrent networks operating under a limited energy budget in predictive environments. In addition to category-specific inhibition and increasingly sharpened predictions with time, we observed that the RNNs self-organize into mainly two distinct unit populations: error and prediction units. Beyond these processes, we observe two distinct mechanisms of prediction: a faster inhibitory loop among error units and a slower predictive loop that enables RNNs to integrate information over longer timescales and, therefore, generate increasingly fine-grained predictions. This observation can be interpreted as a rudimentary form of hierarchical self-organization in which the predictive units can be viewed as a higher-order cortical population operating on longer timescales and the error units as a lower-order cortical population operating on shorter timescales. This interpretation is consistent with hierarchical predictive coding architectures^18^^,^^22^ as well as the organization of the brain in terms of a hierarchy of timescales.^57^^,^^58^ The phenomenon of specialization in the circuit has also been demonstrated to be a useful notion by Zeldenrust et al.,^59^ who analytically derived a class of spiking recurrent predictive coding networks with neural heterogeneity and have shown that these networks can better explain away prediction errors than homogeneous networks. This may indicate that neural heterogeneity and specialization are integral parts of neural systems that have to be efficient in predictive environments.

Our loss function is computed from the network preactivation and, therefore, fully depends on the input drive and the recurrent drive. This implies an optimal solution in which the recurrent drive corresponds to the negative input drive. The network could therefore be seen as optimizing for the negative target, and, therefore, in this context, there is little to no difference between energy minimization and prediction of network input.

Although it is true that minimizing the preactivation is equivalent to predicting the (negative) input drive, this does not necessarily imply a predictive coding framework. In particular, predictive coding as a framework consists of a functionally distinct population of units interacting in a hierarchical fashion.^54^ Thus, the loss function minimized, despite its functional relationship to prediction, does not build in these assumptions of structure. This allows us to conclude that our loss function, although intimately linked to prediction, does not assume the predictive coding framework and that this is instead an emergent property.

One might wonder why we opted for RNNs over feedforward networks, given their popularity in brain data modeling. We believe that the time-varying nature of the network input requires a network architecture that can take time into account during its computation through a form of recurrence. In principle, this can be implemented in feedforward networks by temporally unrolling an equivalent RNN. However, without weight sharing, such a network would not be able to implement lateral or top-down information flow and would be expensive in terms of parametric complexity.

This work builds on a number of studies investigating the relationship between predictive systems, environments, and their effects on efficiency. From a thermodynamics perspective, Still et al.^34^ demonstrated that a system with memory exposed to a stochastic signal must be predictive to operate at maximal energy efficiency. Candadai and Izquierdo^35^ showed, information theoretically, that predictable environments produce neural networks that exhibit predictive information. In the work by Sengupta et al.,^60^ the minimization of thermodynamic energy was linked to information processing and efficiency using the Jarzynski equality. This formulation highlights the deep connection between the thermodynamic energy and the computational cost of (Bayesian) belief updating. In this instance, the authors were able to show that the thermodynamic equilibrium, which minimizes thermodynamic free energy, coincides with the minimum of variational free energy that underwrites approximate Bayesian inference.^31^^,^^32^^,^^33^^,^^36^ This is important because predictive coding can be cast as Bayesian filtering (e.g., Kalman filtering), which entails a minimization of variational free energy.^61^ This variational free energy is also known as an evidence lower bound and is the objective function used in variational autoencoders.^32^

Although we have framed the choice of the preactivation energy function in terms of thermodynamic free energy, we could have also chosen an information theoretic cost function based on variational free energy. Interestingly, variational free energy can also be derived in terms of minimum message length or minimum description length in the setting of Kolmogorov complexity and Solomonov induction and related formulations.^31^^,^^62^^,^^63^ In turn, this leads to formulations of universal computation that speak again to the minimization of (variational free) energy in affording the most compressed representation of some input. This was the original motivation for linear predictive coding in the 1950s^64^ and fits very comfortably with the formalism presented in this work.

On a neural level, it was demonstrated that tightly balanced excitation-inhibition can be understood as neural networks efficiently coding information, with membrane potentials of neurons interpretable as a prediction error of a global signal.^65^^,^^66^^,^^67^^,^^68^ Masumori et al.^69^ demonstrate that a spiking neural network, solely trained based on spike timings, learns to predict temporal sequences, proposing that predictive coding arises to avoid stimulation of biological networks. This current work extends this body of evidence by demonstrating that the framework for predictive coding can emerge in recurrent networks that are trained for a simple consideration of energy efficiency, reproducing functional components of predictive coding in a biologically inspired network setup. To concretely link the impact of our loss function to energy efficiency in terms of action potentials and synaptic weights, we derive the impact of noise and input variability (zero mean but with arbitrary distribution) as aiding in minimization of the size of the synaptic weights used to minimize the network’s activity and precluding a solution where synaptic weights are large but cancelling one anothers’ network impact.

The current work, because of its reliance on smaller-scale datasets (MNIST and CIFAR-10), represents a proof of concept. The reliable findings across two separate datasets and the aforementioned findings regarding the link between prediction and energy efficiency indicates, however, that the observed results are likely more general. Larger categorical datasets, such as CIFAR-100 or ImageNet, would increase the complexity of the problem by the larger number of categories available. This would allow training and testing on longer sequences. We do not see principled reasons why these longer sequences should not yield a similar separation into prediction and error units. We also must take note of the fact that our loss function does not cover all metabolic sources in the brain, such as the maintenance associated with longer wire lengths,^70^ which can induce more energy constraints on the model. It will be interesting for future work to investigate the interplay of both sources of energy cost, information transfer and wiring length, in a joint setting.

Given that we hypothesized that energy efficiency is sufficient for the basic components of the predictive coding framework to emerge, we restricted ourselves to a single-layer recurrent network in which units can freely connect to each other. Future work would entail extending the current model to a multilayer network so that communication between prediction and error units between layers can be investigated in more detail. In terms of model details, future work could also explore energy efficiency in spiking network models where membrane voltages and spike times must be considered because the current set of results relies on a rate-coded neuron model. How a predictive system should optimally process differing spatial and temporal scales of dynamics and deal with unpredictable but information-less inputs (e.g., chaotic inputs and random noise) are key areas for future consideration.

Our current set of findings suggests that predictive coding principles do not necessarily have to be hard-wired into the biological substrate but can be viewed as an emergent property of a simple recurrent system that minimizes its energy consumption in a predictable environment. We have shown here that minimizing unit preactivations implies minimizing unit activity and synaptic transmission. This observation opens interesting avenues of research into efficient coding and neural network modeling.

## Experimental procedures

### Resource availability

#### Lead contact

The lead contact is A.A. (abdullahi.ali@donders.ru.nl).

#### Materials availability

There are no newly generated materials.

### Data generation

#### MNIST

The input data are sequences of images drawn from the MNIST database of handwritten digits.^50^ Images are of size 28×28 with pixel intensities in the range [0,1]. There are 60,000 images in the training set and 10,000 images in the test set. Each set of images can be divided into 10 categories; one for each digit. The frequency of each digit category in the dataset varies slightly (frequencies lie within 9%–11% of the total dataset (70,000 samples)). Sequences are generated by choosing random digits as starting points. Digits from numerically subsequent categories are then randomly sampled and appended to the sequence until the desired sequence length is reached. The categories are wrapped around so that, after category “nine,” the sequence continues from category “zero.” The sequence length can be chosen in advance, but all sequences are constrained to have the same length (in our simulations, we take a sequence length of 10). The sequences are organized into batches. The images are drawn without replacement, and the process is stopped when there are no image samples available for the next digit. Incomplete batches, in which there are no remaining image samples to complete a sequence, are discarded.

#### CIFAR-10

The input data are sequences of images drawn from CIFAR-10, a labeled subset of the tiny image database.^71^ CIFAR-10 consists of 10 classes. These classes are as follows: airplanes, cars, birds, cats, deer, dogs, frogs, horses, ships, and trucks. Images are colored and of size 32×32×3 with pixel intensities in the range [0,1]. There are 50,000 images in the training set and 10,000 images in the test set. Each set of images can be divided into 10 categories; one for each class. The frequency of each category in the data set is exactly 6,000. Sequences are generated by choosing random images as starting points. Images from numerically subsequent categories are then randomly sampled and appended to the sequence until the desired sequence length is reached. The categories are wrapped around so that, after category “nine,” the sequence continues from category “zero.” The sequence length can be chosen in advance, but all sequences are constrained to have the same length (in our simulations, we take a sequence length of 10). The sequences are organized into batches. The images are drawn without replacement, and the process is stopped when there are no image samples available for the next class. Incomplete batches, in which there are no remaining image samples to complete a sequence, are discarded.

### RNN architecture and training procedure

We created a fully connected RNN consisting of 784 units for MNIST and 3,072 units for CIFAR-10. Each unit is driven by exactly one input pixel, which means that the number of units exactly matches the number of pixels in the image. The equations that determine the RNN dynamics are

(Equation 1) $$p_{t}=Wh_{t-1},$$

(Equation 2) $$a_{t}=p_{t}+x_{t},$$

(Equation 3) $$h_{t}=f\left(a_{t}\right),$$

where $x_{t}\in R^{N}$ denotes the input drive, $a_{t}\in R^{N}$ denotes the preactivation, $W\in R^{N\times N}$ denotes the recurrent weight matrix of the RNN (the only learnable parameters), $p_{t}\in R^{N}$ denotes the recurrent feedback, and $h_{t}\in R^{N}$ denotes the unit outputs (in our study, f are ReLU non-linearities). The subscripts t and t−1 refer to the discrete (integer) timestep of the system because all of these variables (aside from the weights) are iteratively updated. The weight matrix is uniformly initialized in [−1,1] and scaled by $N^{-\frac{1}{2}}$; i.e., proportionally scaled by the number of units in the weight matrix.

The objective of minimizing energy is captured through the following loss function:

(Equation 4) $$\ell =\frac{1}{NT}\sum_{t=1}^{T}\|a_{t}{\|}_{1},$$

where T is the number of time steps, N is the number of units, and $a_{t}$ is the preactivation of the units when processing the t -th element (or timestep) in a given sequence. We trained 10 model instances for 200 epochs on MNIST and 10 model instances for 1,000 epochs on CIFAR-10 with batch size 32 (32 sequences per batch) with the Adam optimizer ($\beta_{1}=0.9$, $\beta_{2}=0.999$, learning rate = 0.0001).^72^ Model training differed in weight initialization and training sequence. We tested the same 20 model instances (lesioned models in Figure 4A and Figure 5C) but with unit activity $h_{t-1}$ masked so that the feedback-driven units did not affect the computation of $a_{t}$. No extensive hyperparameter search was performed.

In addition to the models trained with the preactivation loss, we also trained two baseline models (10 instances each) with equivalent architectures and training procedures with different losses. The first baseline was trained with a loss on the unit activity post non-linearity (i.e., network activity):

(Equation 5) $$\ell_{b1}=\frac{1}{NT}\sum_{t=1}^{T}h_{t},$$

The second baseline was trained with the post-non-linearity loss and L2 regularization on the weights

(Equation 6) $$\ell_{b2}=\frac{1}{NT}\sum_{t=1}^{T}\left(h_{t}+\lambda \left\|W\right\|\right),$$

Here λ controls the relative impact of the weight regularization and is picked (through a search) to be as close as possible to the energy consumption of the preactivation loss model (here λ=3708). The models were trained using PyTorch 1.5.0 and Python 3.7.7.

### Determination of energy consumption

To benchmark how well the model minimizes energy consumption, we use the following measure for energy consumption for a particular sample m on a particular time point t:

(Equation 7) $$E_{t}^{m}=\alpha_{1}\bar{h_{t}^{m}}+\alpha_{2}\bar{s_{t}^{m}},$$

where

(Equation 8) $$s_{t}^{m}=\left|h_{t}^{m}\right|⊗\left|W\right|,$$

$\alpha_{1},\alpha_{2}$ are constants that weigh the impact of activity and synaptic transmission (in this work, $\alpha_{1}=\frac{1}{3}$, $\alpha_{2}=\frac{2}{3}$) and $\bar{h_{t}^{m}}$, $\bar{s_{t}^{m}}$ denote the average network activity and network synaptic transmission for m at time point t. The mean energy consumption for a network at time point t over all samples in the test data set will be

(Equation 9) $${\bar{E}}_{t}=\sum_{m=1}^{M}\alpha_{1}\bar{h_{t}^{m}}+\alpha_{2}\bar{s_{t}^{m}}.$$

### Determination of prediction and error units

To determine whether units are predictive, we record their median preactivations for each category at the final time step of a sequence, when the dynamics of the network are most stable. We construct 99 % CIs around this median. To obtain a 99 % CI, we first compute the MAD around the category median preactivation. Supposing $\mu^{\frac{1}{2}}(a_{i}^{\left(c\right)})$ is the category median preactivation at the final time point for a particular unit i, we can compute the MAD for a particular category and unit as follows:

(Equation 10) $$MAD_{i}^{\left(c\right)}=\mu^{\frac{1}{2}}\left(\left|a_{i,m}^{\left(c\right)}-\mu^{\frac{1}{2}}\left(a_{i}^{\left(c\right)}\right)\right|\right),$$

where $a_{i,m}^{\left(c\right)}$ is the preactivation of unit i for an arbitrary sample m of category c. This means that you can find the MAD for a particular unit and category by taking the median of the absolute deviations of the unit sample preactivations from the median preactivation.

CIs can only be analytically calculated for Gaussian distributed random variables using the standard deviation around the mean, so we have to use an approximation. We can do this by converting the obtained MADs to pseudo-standard deviations^73^ in the following way:

(Equation 11) $${\hat{\sigma }}_{i}^{\left(c\right)}=1.4826\cdot MAD_{i}^{\left(c\right)},$$

where ${\hat{\sigma }}_{i}^{\left(c\right)}$ is the pseudo-standard deviation around the median preactivation for unit i and category c, and 1.4826 is the scaling constant for Gaussian distributed variables.

The 99 % CI will then be

(Equation 12) $$CI_{i}^{\left(c\right)}=\left[\mu^{\frac{1}{2}}\left(a_{i}^{\left(c\right)}\right)-2.576{\hat{\sigma }}_{i}^{\left(c\right)},\mu^{\frac{1}{2}}\left(a_{i}^{\left(c\right)}\right)+2.576{\hat{\sigma }}_{i}^{\left(c\right)}\right].$$

where 2.576 is the *Z* score that determines the bounds of the CI. A unit i will be identified as a prediction unit when $0\notin CI_{i}^{\left(c\right)}$ for at least one c.

To determine whether units signal error, we provide the networks with additional sequences that have a late-time-point (in this work, t=8) distractor (an image that breaks the order of the sequence) and record the unit responses (i.e., unit outputs post non-linearity). For each unit i and class c, a distribution over responses for distractor sequences $P_{id}^{\left(c\right)}\left(r_{id}^{\left(c\right)}\right)$ is constructed and compared with $P_{i}^{\left(c\right)}\left(r_{i}^{\left(c\right)}\right)$, the response distribution for normal sequences, where

(Equation 13) $$P_{i}^{\left(c\right)}\left(r_{i}^{\left(c\right)}\right)=N_{i}^{\left(c\right)}\left(r_{i}^{\left(c\right)};\mu_{i}^{\left(c\right)},{\sigma_{i}^{\left(c\right)}}^{2}\right),$$

(Equation 14) $$P_{id}^{\left(c\right)}\left(r_{id}^{\left(c\right)}\right)=N_{id}^{\left(c\right)}\left(r_{id}^{\left(c\right)};\mu_{id}^{\left(c\right)},{\sigma_{id}^{\left(c\right)}}^{2}\right).$$

Unit i is determined to be signaling error for class c when

(Equation 15) $$\frac{\left|\mu_{i}^{\left(c\right)}-\mu_{id}^{\left(c\right)}\right|}{\sqrt{{\sigma_{i}^{\left(c\right)}}^{2}+{\sigma_{id}^{\left(c\right)}}^{2}}}>2.576,$$

that is, we can say with 99% confidence that the distributions are different.

### Procedure for prediction unit lesions and control lesions

The procedure for the lesion experiments on prediction units is as follows: using the method described in “Determination of prediction and error units,” prediction units were detected for all digit classes. Subsequently, the set of activations and outputs of all prediction units was set to zero during inference to ensure that their activity cannot affect the other units in the network. A similar procedure was employed for the control lesion experiments. In this case, however, random sets of non-prediction units in the network were lesioned. The number of lesioned units in the control condition was set to be equal to the original number of prediction units lesioned.

### Calculating postsynaptic drive dynamics of prediction and error units

To calculate the postsynaptic drive dynamics of prediction and error units, we first determined the prediction and error units (separately for each network instance). For each prediction and error unit, we calculate their synaptic transmission for each sequence and time point. The resulting matrix is then averaged over the number of sequences, yielding a mean postsynaptic drive curve for each prediction and error unit. The corresponding curves, shown in Figure 4C, indicate the average synaptic transmission originating from prediction and error units, averaged across network instances together with 95% Cis, obtained via bootstrapping.

## Data Availability

All data and analysis code along with instructions are available at https://osf.io/c57d4/(https://doi.org/10.17605/OSF.IO/C57D4).

## Supplemental information

Document S1. Figures S1–S5

Document S2. Article plus supplemental information

## Footnotes

Supplemental information can be found online at https://doi.org/10.1016/j.patter.2022.100639.
