# Supplementary material for: Predictive coding is a consequence of energy efficiency in recurrent neural networks
Source: Patterns (N Y). 2022 Nov 23;3(12):100639. doi: 10.1016/j.patter.2022.100639 (PMC9768680; doi:10.1016/j.patter.2022.100639)
Supplement: Document S1. Figures S1–S5 [file mmc1.pdf]

Patterns, Volume *3*

Supplemental information

Predictive coding is a consequence of energy efficiency in recurrent neural networks

Abdullahi Ali, Nasir Ahmad, Elgar de Groot, Marcel Antonius Johannes van Gerven, and Tim Christian Kietzmann

Supplemental figures

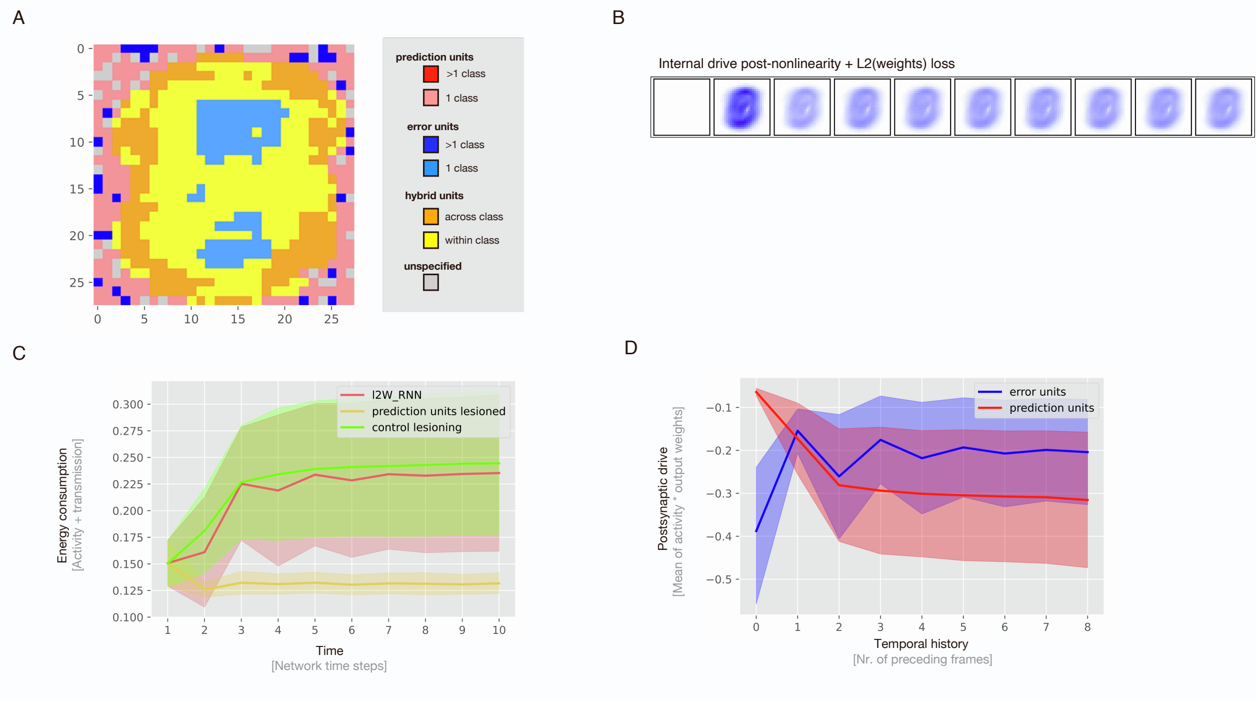

Figure S1: Results of models trained to minimise activity with and L2-regularisation term on the weights. The network fails to learn sharp predictions (**B**), as well as produce functionally distinct prediction and error units (**A**,**C**, **D**). Regularisation-term determined through a linear search over the parameter and selecting the model that comes closest to the preactivation loss networks in terms of performance

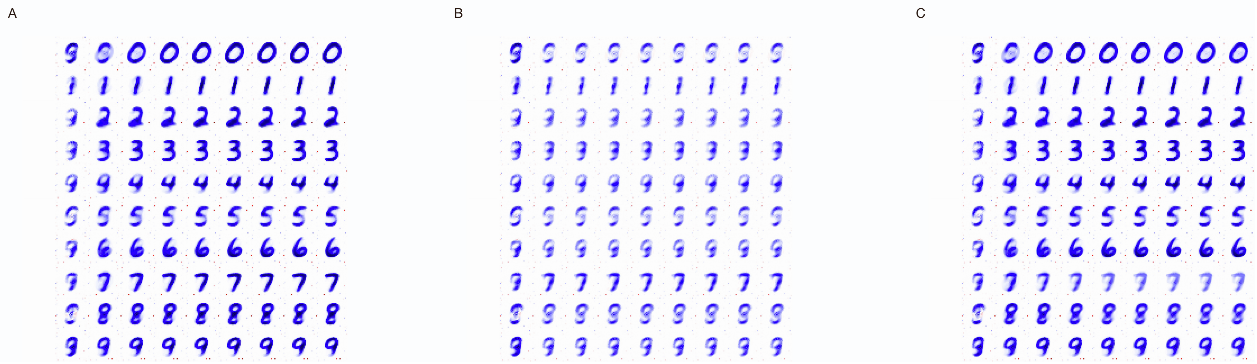

Figure S2: **A:** Recurrent feedback ($\mathbf{p_t}$) in the RNN for different input categories at different points in time. Each row shows the recurrent feedback at the step with a specific input category will be expected. Each column shows the recurrent feedback after a different number of preceding images in the sequence. The inhibitory effect (i.e. predictions) gets more pronounced as sequences progress. **B:** Recurrent feedback ($\mathbf{p_t}$) in the lesioned RNN model for different input categories at different points in time. The inhibitory effect with lesioned prediction units is weak and remains constant throughout the sequence.Recurrent feedback ($\mathbf{p_t}$) when the lesioning is targeted to a particular class (in this instance digit 7). The lesioning now primarily affects the digit 7 class while having little effect on the other classes. This means that category-specific predictions do develop in the network.

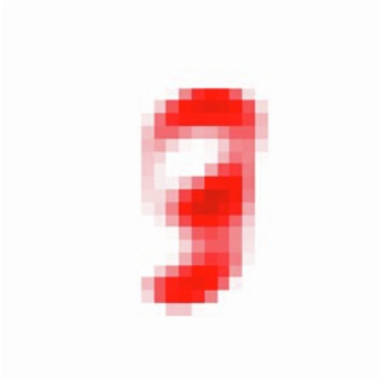

Figure S3: Median image of the MNIST dataset. This image is obtained by calculating the median intensity for each pixel across each sample in the training data set.It essentially represents what the median digit of the entire training data looks like.

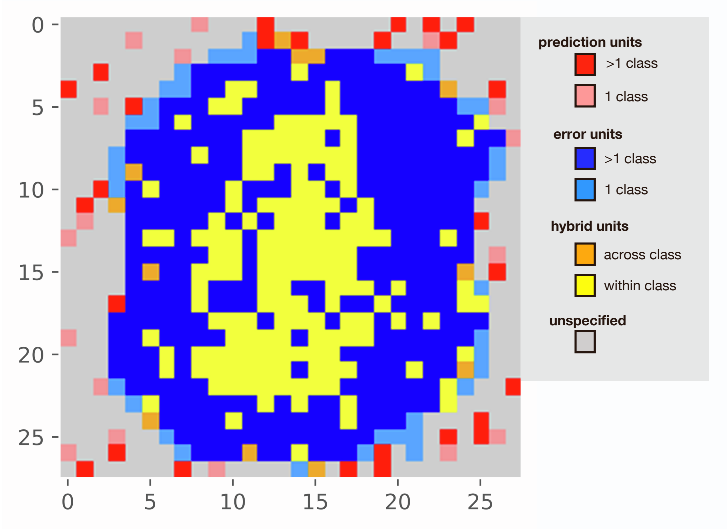

Figure S4: Topographic distribution of prediction and error units as determined for an untrained network. The prediction and error units have much more overlap compared to the trained network as can be seen by the large number of hybrid units. The map is generated with the same parameters as the map in figure 3 of the manuscript.

Supplemental Experimental Procedures

This supplement provides a derivation for the claim in the main manuscript that minimising preactivation leads to smaller weights and thus synaptic transmission. We do this by analysing what weight changes are induced in the case where noise causes predictions to deviate from the optimal predictions.

In this work we apply an L1 cost function to the preactivations of our units in mini batches, such that for a network of N units, and a given mini batch b consisting of M samples,

$$\ell^b = \frac{1}{M} \sum_{M}^{m} \sum_{N}^{n} |x^b_{mn} - p^b_{mn}| \,, \tag{1}$$

where ℓ^b is the loss function measured for mini batch b. For simplicity's sake, let us assume a batch size of one and consider a single unit within our network. This allows us to write this loss as:

$$\ell^b_n = |x^b_n - p^b_n| \,, \tag{2}$$

where x_n are the input drives to each unit, indexed n, and p_n is the recurrent network feedback which is learned in order to minimise this loss.

However, in this network we train the system to minimise the loss of prediction with some history of dynamics. Assuming that our history of dynamics are consistent but with some batch-dependent error ϵ, our recurrent prediction simplifies to:

$$\ell^b_n = |x^b_n - (W(h^b + \epsilon^b))_n| \,. \tag{3}$$

We could now plot the set of losses across all of these batches such that we would observe a distribution of losses. By carrying out SGD to minimise our loss, we would minimise the mean and the variance of this distribution.

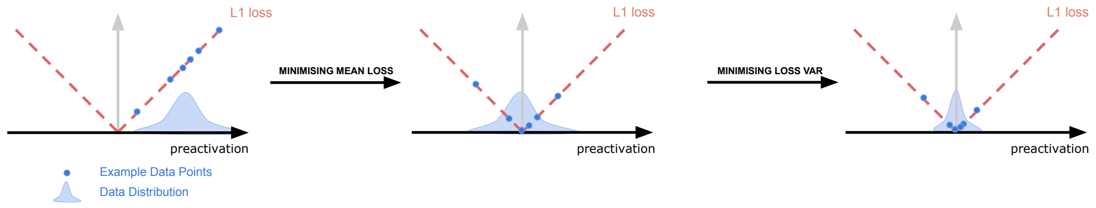

Figure S5: Visualisation of what happens to the loss after training. X-axis shows the preactivation for a particular data point, y-axis shows the associated loss. We can construct a distribution over the losses as well as plot the loss function to see how far the current networks performance is from optimal. Minimising the mean will not be enough to reach the optimal solution, Since the variance also contributes to the loss of network. Thus SGD will minimise the variance and the mean

Average loss

The average loss can be computed as an expectation over samples such that

$$\langle \ell^b_n \rangle_b = \mu_\ell = \frac{1}{B} \sum_{B}^{b} |x^b_n - W(h + \epsilon^b)| \tag{4}$$

$$= \frac{1}{B} \sum_{B}^{b} |x^b_n - Wh| + H_b| - W\epsilon^b| \,, \tag{5}$$

where H_b is an signed variable (-1/+1) depending upon whether the contribution of noise increases or decreases the magnitude of the loss, $H_b = \text{sgn}(x^b_n - Wh)\ \text{sgn}(-W\epsilon^b)$ assuming no switches in signs are introduced by the noise.

Now, in computing this average, we can say that if the noise is uncorrelated (random) w.r.t. the activity and weights and has zero mean (unbiased), that it will average out such that $\langle H_b |W\epsilon^b| = 0.0 \rangle$ and thus

$$\mu_\ell = \frac{1}{B} \sum_{B}^{b} |x^b_n - Wh| \,. \tag{6}$$

Hence, in order to minimise our average loss, we would attempt to produce prototypical predictions.

Variance of loss

Now considering the variance of our loss

$$Var(\ell_n^b) = \frac{1}{B}\sum_B^b (|x_n^b - W(h+\epsilon^b)| - \mu_\ell)^2 \tag{7}$$

$$= \frac{1}{B}\sum_B^b (|x_n^b - Wh| + H_b| - W\epsilon^b| - \mu_\ell)^2 \tag{8}$$

$$= \frac{1}{B}\sum_B^b ((|x_n^b - Wh| - \mu_\ell) + H_b| - W\epsilon^b|)^2 \tag{9}$$

$$= \frac{1}{B}\sum_B^b (|x_n^b - Wh| - \mu_\ell)^2 + (H_b| - W\epsilon^b|)^2 + 2H_b| - W\epsilon^b|(|x_n^b - Wh| - \mu_\ell) \tag{10}$$

$$\approx \frac{1}{B}\sum_B^b (|x_n^b - Wh| - \mu_\ell)^2 + (H_b| - W\epsilon^b|)^2 \tag{11}$$

$$\approx Var(Signal) + Var(Noise), \tag{12}$$

where we now have three terms (see eq 6), one term which is the variance of the noiseless predictions, a measure of the variance of noise term (scaled by W) and a cross-term correlating the noise term. Note that the correlated noise term involves multiplying two random variables, each with a mean of zero, thus we ignore this term as its expected value is zero. Finally, we are therefore left with terms of the signal and noise variance. The signal variance cannot be reduced since it is inherent to the data. The noise term however, scales with W, thus to reduce the variance caused by our noise term the weights, W, would be reduced. Note that the noise here refers to noise in your prediction, i.e. an uncertainty about the upcoming stimulus which is the case in real data.
